# Cytotoxicity and genotoxicity of urban particulate matter in mammalian cells

**DOI:** 10.1093/mutage/gev025

**Published:** 2015-06-25

**Authors:** Audrey F. Dumax-Vorzet, M. Tate, Richard Walmsley, Rhod H. Elder, Andrew C. Povey

**Affiliations:** Centre for Occupational and Environmental Health, Centre for Epidemiology, Institute of Population Health, Faculty of Medical and Human Sciences, The University of Manchester, Ellen Wilkinson Building, Manchester M13 9PL, UK,; ^1^Gentronix Ltd, BioHub at Alderley Park, Alderley Edge, Macclesfield, Cheshire, SK10 4TG, UK,; ^2^School of Environment and Life Sciences, University of Salford, Cockcroft Building, Salford M5 4WT, UK

## Abstract

Ambient air particulate matter (PM)-associated reactive oxygen species (ROS) have been linked to a variety of altered cellular outcomes. In this study, three different PM samples from diesel exhaust particles (DEPs), urban dust standard reference material SRM1649a and air collected in Manchester have been tested for their ability to oxidise DNA in a cell-free assay, to increase intracellular ROS levels and to induce CYP1A1 gene expression in mammalian cells. In addition, the cytotoxicity and genotoxicity of PM were assessed using the 3-(4,5-dimethylthiazol-2-yl)-2,5-diphenyltetrazolium bromide assay and alkaline comet assay, respectively. All PM samples catalysed the Fenton reaction in a cell-free assay, but only DEP resulted in the generation of ROS as measured by dichlorodihydrofluorescein diacetate oxidation in mammalian cells. However, there was no evidence that increased ROS was a consequence of polycyclic aromatic hydrocarbon metabolism via CYP1A1 induction as urban dust, the Manchester dust samples but not DEP-induced CYP1A1 expression. Urban dust was more cytotoxic in murine embryonic fibroblasts (MEFs) than the other PM samples and also induced expression of GADD45a in the GreenScreen Human Cell assay without S9 activation suggesting the presence of a direct-acting genotoxicant. Urban dust and DEP produced comparable levels of DNA damage, as assessed by the alkaline comet assay, in MEFs at higher levels than those induced by Manchester PM. In conclusion, results from the cytotoxic and genotoxic assays are not consistent with ROS production being the sole determinant of PM-induced toxicity. This suggests that the organic component can contribute significantly to this toxicity and that further work is required to better characterise the extent to which ROS and organic components contribute to PM-induced toxicity.

## Introduction

The health effects of particulate matter (PM) have been documented widely (1,2) and there is increasing evidence to suggest that PM-induced toxicity is a result of the generation of reactive oxygen species (ROS) (3–5). PM can cause the formation of ROS by a variety of mechanisms including the initiation and stimulation of the inflammatory response both *in vivo* and in macrophage-derived cells in culture (6,7), and the catalysis of H_2_O_2_ degradation by PM-associated transition metals resulting in hydroxyl radical formation via the Fenton reaction (8–10). The ability of transition metals to generate ROS depends on their abundance, bioavailability and oxidation state in PM. In addition to ROS generation and the subsequent induction of oxidative stress in cells, organic chemicals can be adsorbed onto the surface of PM and contribute further to its toxicological properties. Indeed, both the organic and aqueous extracts of PM can induce DNA strand breaks in cellular systems (11). Organic extracts have been shown to contain a range of potent carcinogens and mutagens such as polycyclic aromatic hydrocarbons (PAHs) (12). Cytochrome P450 (CYP)-mediated PAH metabolism, through either the *o*-quinone pathway or naturally occurring quinoid redox cycling to quinone and semi-quinone via one-electron oxidation, may also be an important source of ROS (13,14).

Studies in mammalian cells have shown that exposure to PM can result in increased cell death (6,15,16), increased levels of DNA strand breaks, oxidative base damage and PAH-DNA adducts (15,17–21), together with an increased mutation frequency (22,23) and genetic rearrangements (24,25). Urban PM can induce a dose-dependent decrease in mammalian cell viability accompanied by an increase in DNA damage (6,26,27). The magnitude of the observed effect depends not only on the PM size (28) but also the chemical and gravimetrical composition of PM (6,29,30). For example, a multi-centre European study has clearly demonstrated that the *in vitro* genotoxicity of organic extracts of PM_10_ particles can vary with PM source (31). Given the known health effects of PM and the variability in PM content with source, we have investigated the cytotoxicity and genotoxicity of PM samples collected from a busy urban thoroughfare in Manchester (UK) and compared them with standard urban dust particulate sample [standard reference material (SRM1649a)] and diesel engine particles (DEPs). We also examined the role of ROS in the cellular toxicity induced by these PM samples.

## Materials and methods

### Materials

Two portable urban dust samplers (Rotheroe & Mitchell Ltd, UK) were placed on the first floor of the Student Union building, Oxford Road, Manchester, UK. The pump units were indoors while the collection units were placed outdoors through an open window. Sampling was carried out during the summer of 2008 (13 August–10 September) and winter of 2009 (3–28 February) and total suspended particulate matter (TSP) was collected from 8:00 to 18:00 weekdays on polytetrafluoroethylene (PTFE) filters. Used filters were collected every night and equilibrated overnight at room temperature before being weighed and stored at −20°C. Clean filters were similarly equilibrated, weighed and placed every morning on the inlet of the samplers.

An estimated 930 m^3^ (in 20 weekdays) and 710 m^3^ (in 14 weekdays) of urban air were filtered and ~14 and 16mg of TSP were collected in Manchester in the summer and winter, respectively. TSP was then extracted using a previously published method with minor alterations (32). Briefly, PTFE filters were cut into quarters, pre-wetted with 100% ethanol, immersed in 12ml of American Chemical Society (ACS) grade water per filter (Sigma-Aldrich, UK), then sonicated for 30–45min (Cavitator ultrasonic cleaner, Mettler Electronics Corp., Sarose Scientific Instruments, UK). The filter pieces were then removed and the aqueous solution was lyophilised. Different batches of dried TSP were combined to obtain homogeneous winter and summer TSP samples and subsequently diluted to 20mg/ml in dimethylsulfoxide (DMSO) and kept at −20°C in the dark until used. Approximately 48 and 78% of the summer and winter TSP samples, respectively, were recovered. To control for intrinsic PTFE filter effects, blank PTFE filter extracts were prepared similarly. DEP was collected from a new race car diesel engine without catalytic filter, tested on an industrial test bed through a range of conditions including load and acceleration (gift from Dr A. Hirst, University of Manchester). Urban PM SRM1649a was purchased from the US National Institute of Standard and Technology (NIST). All other reagents were from Sigma-Aldrich, UK.

### Environmental scanning electron microscope-energy dispersive X-ray (EDX) analysis

To ensure that the particles analysed were representative of the whole PM sample (and not only of composition on a few sampling days), PM (~40 µg) was diluted in 2.5ml ACS water and filtered through a Pop-Top filtration device (GE Healthcare) loaded with a polycarbonate (PC, Nucleopore) filter. The latter was air-dried overnight and kept at −20°C. The carbon-coated PC filter was placed in the chamber of the environmental scanning electron microscope (ESEM–EDX XL-30, Phillips). The distance between the filter and the gaseous secondary electron detector was set at 10mm and the water vapour pressure in the microscope chamber at 0.5 Torr. The acceleration voltage for the electron beam was set at 15 keV. EDAX–energy dispersive X-ray spectroscopy (EDS) system and Genesis software (Ametek, Inc.) allowed for automatic particle detection along with the identification and relative quantification of different chemical elements based on the X-ray energy spectra emitted by the analysed particle. Individual particles were selected automatically and analysed by the EDAX software on >70 preset, non-adjacent analytical fields spread throughout the analysed filter for each air pollution sample. Data for each particle were then verified manually so as to exclude artefacts. Compositional data for >286 particles were collected for each PM sample using the automatic particle analysis software. Mean relative elemental composition of the different particles analysed in the same PM sample (and associated SEM) was calculated for each PM sample. Particle size of the PM sample was measured for each particle using the EDAX software.

### Plasmid scission assay

The ability of PM to cause DNA strand breaks *in vitro* was investigated by the plasmid scission assay (PSA). PM (up to 0.1mg/ml), FeSO_4_ and 0.8mM H_2_O_2_ (individually or in combination) were added to 80ng pBR322 plasmid in 10mM Tris–HCl, pH 8.5. Samples were incubated for 6h at 20°C after which the different plasmid DNA conformations were separated by agarose gel electrophoresis. Ethidium bromide-bound DNA was visualised using a Typhoon 9400 multimode imager (GE Healthcare) and the intensity of supercoiled, relaxed and linear plasmid in each lane was quantified using ImageQuant™ software (GE Healthcare). The level of damaged plasmid (i.e. relaxed and linear) was calculated as a proportion of the combined intensity of all three plasmid forms. Three to five replicates were carried out and mean damaged plasmid level (and associated SEM) for each PM dose was calculated.

### 3-(4,5-Dimethylthiazol-2-yl)-2,5-diphenyltetrazolium bromide assay

Murine embryonic fibroblasts (MEFs) were used in this study because we have used them previously to examine toxicity of alkylating agents (33) and ROS (34) and they have been shown to have the metabolic competence to activate a number of environmental carcinogens, including benzo(a)pyrene, a PAH potentially present in PM (35).

MEFs were immortalised by continuous growth in 20% O_2_ at 37°C. Cells surviving after ~10 passages were assumed to have undergone spontaneous immortalisation. Subsequent cell culture was performed under 3% O_2_. All cells were grown in DMEM:F12 (Invitrogen, UK) supplemented with 10% (v/v) heat-inactivated foetal calf serum (PAA Biotechnology, UK), 2mM L-glutamine and 0.11M NaHCO_3_ (Invitrogen, UK). Approximately 800 MEFs were plated in each well of a 96-well plate and allowed to settle for 24h. The medium was then replaced with serially diluted PM-containing medium (up to 0.1mg/ml) in triplicate wells. After 48h, PM-containing medium was replaced with 0.6mg/ml 3-(4,5-dimethylthiazol-2-yl)-2,5-diphenyltetrazolium bromide (MTT) solution in medium and incubated for 4h. MTT-containing medium was removed and 200 μl DMSO was added. Absorbance at 570 and 690nm (background correction) was measured in each well. Absorbance at 570nm (corrected for background) in triplicate wells was averaged. Cell viability was calculated as a ratio of the corrected 570nm absorbance in control wells. Three to five replicates were carried out and mean cell viability (and associated SEM) at each dose was calculated.

### Intracellular ROS detection assay

An intracellular ROS assay was performed as described by Wang and Joseph (36) with minor alterations. Briefly, 10^4^ MEFs were added to each well of a 96-well plate and left to settle for 24h. The medium was removed and the cells washed with phosphate-buffered saline (PBS) before the addition of 50 µM 2′,7′-dichlorodihydrofluorescein diacetate (DCFH_2_-DA, Invitrogen, UK) in KRH buffer (0.13M NaCl, 5mM NaHCO_3_, 4.8mM KCl, 1.2mM KH_2_PO_4_, 1mM CaCl_2_, 1.2mM MgCl_2_, 2.8mM glucose, 10mM HEPES pH 7.4). Cells were incubated for 30min in the dark at 37°C. DCFH_2_-DA-containing mixture was then removed and the cells were washed with PBS and incubated with H_2_O_2_ (0.8mM) or PM (up to 0.05mg/ml) in KRH buffer in triplicate wells. The fluorescence (λ_ex_ 485nm, λ_em_ 538nm) in every well was measured every 5min for up to 2h at 37°C on a fluorescent plate reader (FluoroSkan Ascent with Ascent, Software™ version 2.4: Thermo Scientific, UK). Changes in fluorescence in treated wells after 45-min incubation (optimum signal-to-noise ratio) were compared with changes in fluorescence during the same time in control wells. Three to five replicates were carried out and the mean change in fluorescence compared to control wells (and associated SEM) for each PM dose was calculated.

### RNA extraction, reverse transcription and reverse transcription–polymerase chain reaction

MEFs (8×10^4^) were added to each well of a 6-well plate and left to settle overnight. Cells were then treated with 20 µg/ml PM (or equivalent volume of DMSO) and incubated for 1–24h. RNA was extracted using the RNeasy kit (Qiagen, UK) according to the manufacturer’s instructions and subsequently treated with 5U RQ1 DNase (Promega, UK). One microgram of RNA was reverse transcribed to cDNA. An equivalent of 25ng reverse-transcribed RNA was used for reverse transcription–polymerase chain reaction (RT–PCR) using primers for CYP1A1 (F: TTCAGTCCCTCCTTACAGCC, R: AAGTCATCTCCCTGCCTCAC) and glyceraldehyde 3-phosphate dehydrogenase (F: AACTTTGGCATTGTGGAAGG, R: ACACATT GGGGGTAGGAACA). RT–PCR products were resolved on a 1.5% agarose gel.

### Alkaline comet assay

MEFs (10^5^) were added to each well of a 24-well plate and allowed to settle for 24h. The medium was replaced with PM-containing medium (up to 0.1mg/ml) and incubated for 3h at 37°C. Cells were trypsinised and diluted in 0.7% low melting point (LMP) agarose (Sigma-Aldrich, UK). Cell viability was verified by trypan blue exclusion. Two drops of LMP-containing MEFs were placed on a microscope slide pre-coated in 1% agarose (Roche, UK). A cover slip was placed on each drop and the slides were incubated at 4°C for 5min after which the cover slips were removed. The slides were then immersed in freshly made lysis buffer (0.2M NaOH, 10mM Tris, 0.12M EDTA, 2.5M NaCl, 1% (v/v) DMSO, 1% (v/v) Triton-X100, pH 10–10.5) for 1h at 4°C. The slides were then washed and submerged in electrophoresis buffer (30mM NaOH, 1mM EDTA, pH > 13) for 40min. Electrophoresis was carried out at 18V for 20min (70–90 mA). After neutralisation in 0.4M Tris–HCl pH 7.5, the slides were air-dried. Prior to analysis, the slides were immersed in water for 30min and stained with 1/10000 SYBRGold (Invitrogen, UK) in 10mM Tris–HCl pH 7.5, 1mM EDTA for 30min in the dark. The slides were washed in water, air-dried and kept in the dark until analysis. Between 18 and 25 fields per slide were randomly photographed using a Nikon Elipse TE 2000-s fluorescent microscope equipped with a Hamamatsu digital camera (Nikon, UK). Per cent DNA in the tail of selected comets was determined using CometScore v1.5 (TriTek Corp., USA). Assays were carried out in triplicate and mean % DNA in the tail (and associated SEM) was calculated for each concentration tested.

### GADD45a-GFP GreenScreen® Human Cell assay

The GADD45a-GFP GreenScreen provides a comprehensive genotoxic hazard assessment: it has a high specificity to genotoxic carcinogens, and its use with and without S9 metabolic activation allows the detection of all mechanistic classes of genotoxicant: mutagens, promutagens, eneugens and clastogens (37). This assay was conducted as described previously (38,39). Briefly, 1.5×10^5^ GenM-T01 cells [reporter cell line expressing green fluorescent protein (GFP)-tagged GADD45a] and GenM-C01 cells (control cell line, in which GFP-GADD45a expression was disabled via deletion of the transcription start site) were plated in each well of 96-well plate in 150 μl TK6 culture medium [RPMI Glutamax, 25mM HEPES, 10% v/v heat-inactivated horse serum (PAA Biotechnology, UK), 1.8mM sodium pyruvate] containing different concentrations of PM (up to 0.4mg/ml) with and without 1% (v/v) S9 rat liver extract. The following controls were used: (i) PM only (autofluorescence); (ii) GenM-T01 + 1% (v/v) DMSO (solvent control); (iii) GenM-T01 + either methylmethanesulfonate (MMS) or cyclophosphamide (positive controls used in the assays without and with S9 extract, respectively); and (iv) culture medium only.

For the assay without metabolic activation, the plate was incubated for 48h at 37°C, 5% CO_2_. Cell viability and GADD45a-GFP expression were assessed by measuring cell density at 620nm and green fluorescence (λ_ex_ 485nm, λ_em_ 535nm), respectively, in each well. Two threshold values were set for cell density and GFP fluorescence: a compound was considered cytotoxic if it decreased cell density by >20% compared to the untreated control. Similarly, a compound was considered genotoxic if GFP fluorescence in the treated cells was at least 1.5 times higher than in untreated control cells.

For the assay with S9 metabolic activation, GenM-T01 and GenM-C01 cell lines were incubated with S9 and PM for 3h at 37°C, 5% CO_2_. S9-containing medium was then removed and the cells were washed in PBS before further incubation for 45h at 37°C in serum-free TK6 culture medium. Before analysis, 25 μg/ml propidium iodide (PI) was added in each well. Plates were incubated at room temperature for 5min before analysis by flow cytometry on a FACSCalibur flow cytometer equipped with a 488-nm argon ion laser. Red fluorescence from PI-stained cells (‘dead’ cells) was detected using a 650-nm longpass filter. GFP green fluorescence from GADD45α-GFP was measured through a 530/30-nm band-pass filter. A minimum of 10000 viable cells (i.e. PI-negative cells) per samples were required for the analysis of GFP fluorescence data. A compound was considered cytotoxic if the cell density was decreased by >20% compared to the untreated control or the proportion of PI-negative cells dropped below 20% of the total cells in the sample. A compound was considered genotoxic if GFP fluorescence in the treated cells was at least 1.3 times higher than in untreated control cells.

### Statistical analysis

One-way analysis of variance was conducted followed by Tukey *post hoc* test using SPSS Statistics version 17.0. All statistically significant results are given with a 95% interval of confidence.

## Results

### Elemental composition and size of PM

The relative elemental composition of PM was assessed by ESEM–EDX (Table 1). Urban dust SRM1649a was markedly different from the other PM samples as it contained the highest amount of sulphate and calcium and the lowest amount of heavy metals (i.e. chromium, vanadium, manganese, cobalt, nickel and zinc). Our ESEM elemental composition data for urban dust 1649a was highly correlated with data obtained previously by NIST using inductively coupled plasma atomic emission spectroscopy (*R* ^2^ = 0.84, *P* = 0.001; data not shown). The elemental composition of urban dust 1649a (determined by NIST or ESEM) did not correlate with any of the other samples analysed. DEP composition was similar to both summer and winter TSP (*R* ^2^ = 0.92, *P* < 0.001 for summer and *R* ^2^ = 0.97, *P* < 0.001 for winter). The elemental composition of summer and winter TSP was also similar (*R* ^2^ = 0.87, *P* < 0.001). The median particle sizes of the different PM were similar and ranged between 1.4 and 2.2 μm with the summer TSP having the smallest median diameter (Table 2).

**Table 1.** Relative elemental composition of PM samples determined by ESEM–EDX

Element	% Mean elemental composition (±SD)			
	Urban dust	DEP	Summer TSP	Winter TSP
Mg	0.9±1.5	1.6±1.2	2.0±1.2	1.7±1.1
Al	3.3±8.1	2.5±1.7	3.8±3.3	3.0±2.2
Si	10.5±21.6	3.8±6.6	14.6±10.7	11.2±14.0
P	2.8±6.0	3.4±2.2	3.2±1.3	3.1±2.0
S	14.9±15.8	4.9±3.0	3.6±1.6	4.9±1.7
Cl	4.3±10.1	3.9±2.5	3.2±1.4	5.5±1.5
K	5.1±4.2	4.5±5.1	4.1±2.2	5.3±2.1
Ca	20.7±22.5	4.5±3.1	6.1±7.6	6.5±4.5
V	2.1±2.6	4.6±3.5	3.9±1.9	5.5±1.8
Cr	2.4±5.5	5.5±4.1	4.2±2.2	5.4±2.2
Mn	1.9±2.5	5.1±4.5	4.4±2.1	5.7±2.0
Fe	9.8±19.4	11.4±14.1	18.9±17.3	11.4±9.8
Co	1.6±3.1	5.9±5.1	5.6±2.8	6.6±2.2
Ni	3.2±7.3	6.6±5.9	6.1±3.3	6.6±2.4
Cu	7.6±19.4	8.4±7.6	8.2±4.1	8.7±3.7
Zn	2.3±4.5	7.8±8.1	8.3±4.6	9.1±3.6

**Table 2.** Comparison of experimental results between the four PM samples

Experimental parameter	Urban dust	DEP	Summer TSP	Winter TSP
Particle size (μm), median (25–75%)	1.7 (1.1–3.0)	2.2 (1.6–3.0)	1.4 (0.8–3.1)	1.9 (1.3–3.2)
Composition correlation	No	Yes	Yes	Yes
Direct ROS generation	−	−	−	−
Catalysis Fenton	+	+	++	+
Intracellular ROS production	−	+	−	−
CYP1A1	+	−	+	+
MTT IC_50_ (μg/ml)	3	15.5	12	13.5
DNA damage^a^	++	++	+	+
GreenScreen assay				
−s9	+	−	ND^b^	ND
+s9	−	−	ND	ND

^a^Strand breaks and alkali-labile sites as assessed by the alkaline comet assay.

^b^Not determined (insufficient sample).

### ROS-generating ability of PM in a cell-free assay

**Figure 1.**
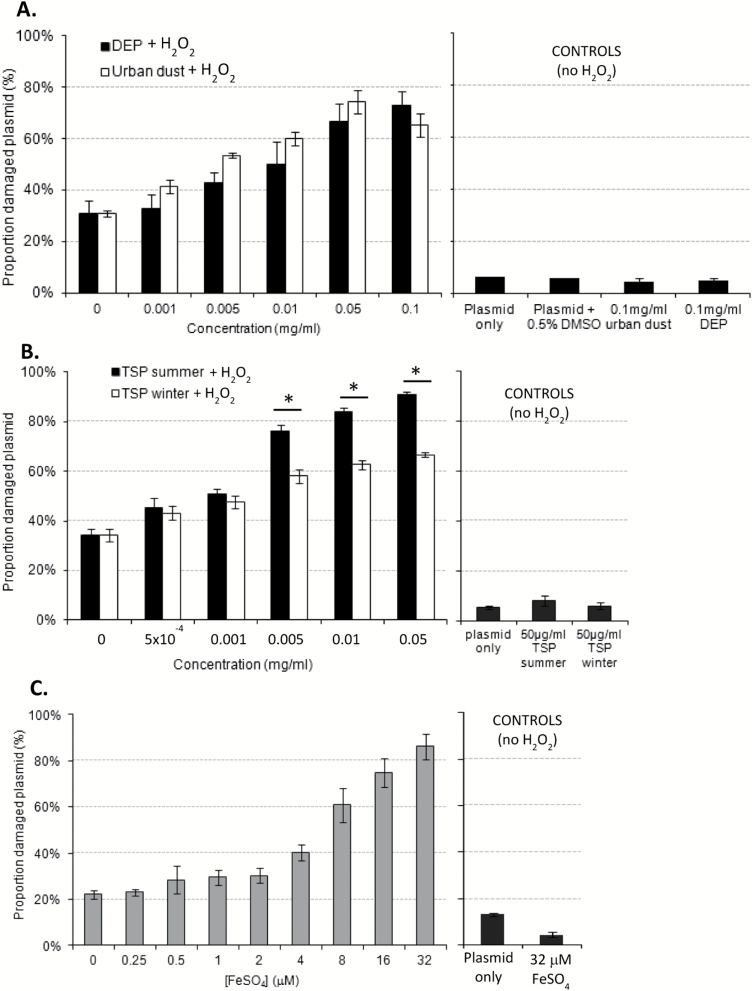
Mean relative plasmid DNA damage induced by urban dust SRM1649a and DEP (**A**), summer and winter TSP (**B**), FeSO_4_ (**C**) in combination with H_2_O_2_. Forty micrograms of pBR322 plasmid was mixed with 0.8mM H_2_O_2_ and PM (A and B) or FeSO_4_ (C) and incubated for 8h at room temperature. H_2_O_2_ was not added in controls, which included: plasmid alone, plasmid with the highest concentration of PM, FeSO_4_ or DMSO. Gel-loading buffer was added to stop the reaction prior to electrophoresis through a 0.6% agarose gel. Ethidium bromide-stained linear, nicked and supercoiled plasmids were visualised with a multimode imager and the intensity of each band was quantified using ImageQuant™. The proportion of damaged plasmid (nicked and linear) in each sample was calculated. Data presented as mean ± SEM (*n* = 4), *P < 0.05 one-way analysis of variance.

The ability of PM to generate DNA-damaging ROS was first investigated in a cell-free system using the PSA. None of the PM samples could generate DNA-damaging ROS in the absence of H_2_O_2_ (see controls, Figure 1A and B). However, in the presence of H_2_O_2,_ a dose–response was observed between PM concentration and relative amount of plasmid damage for all the PM samples studied (Figure 1A and B). DEP caused slightly less plasmid damage than urban dust SRM1649a at most concentrations except for 0.1mg/ml, but these differences were not statistically significant (Figure 1A). Summer TSP caused significantly more plasmid damage than winter TSP at concentrations ranging from 0.005 to 0.05mg/ml (Figure 1B). To investigate whether plasmid DNA damage was caused by ROS generated by the Fenton reaction, plasmid DNA was incubated with H_2_O_2_ and increasing concentrations of FeSO_4_, a potent catalyst of the Fenton reaction (Figure 1C). A dose–response between FeSO_4_ (from 2 to 32 μM) and the relative amount of damaged plasmid showed that Fenton reaction catalysis could lead to plasmid degradation.

### Intracellular level of ROS in MEFs following PM exposure

**Figure 2.**
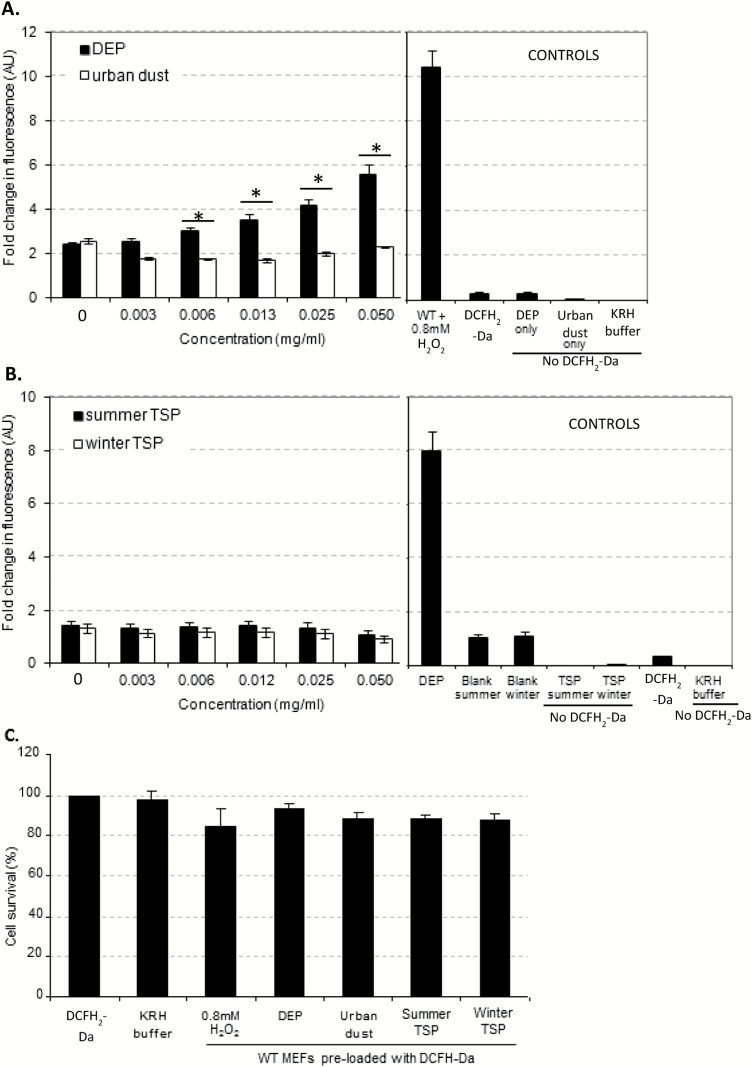
Mean intracellular ROS levels (**A** and **B**) and mean cytotoxicity (**C**) in MEFs following PM exposure. (A and B) MEFs were plated in 96-well plates and allowed to settle for 24h prior to incubation for 30min with 50 µM DCFH_2_-DA in KRH buffer in the dark. DCFH_2_-DA-containing buffer was subsequently removed and 0–50 µg/ml PM was added in triplicate wells [A: urban dust (SRM1649a) and DEP, B: summer and winter TSP]. Oxidation of DCFH_2_-DA to fluorescent DCF was measured every 5min for 2h on a fluorescent plate reader (λ_ex_ 480nm, λ_em_ 530nm). Data presented in (A) and (B) was obtained after 45-min incubation with PM but a similar pattern was observed at each time point. Controls included: cells + DEP or H_2_O_2_ + DCFH_2_-DA (positive controls), DCFH_2_-DA only (without cells), KRH buffer and summer and winter TSP blanks + DCFH-DA. PM (50 µg/ml) in KRH buffer was added in triplicate wells without cells to check for autofluorescence. Results presented as mean ± SEM (*n* = 5), *P < 0.05 one-way analysis of variance (ANOVA). (C) Cytotoxicity of the highest PM dose used was subsequently assessed in DCFH_2_-DA-loaded MEFs, after 2-h incubation using the MTT assay. PM-containing buffer was replaced by 0.6mg/ml MTT and incubated for 2.5h. Formazan precipitate was then dissolved in DMSO before measurement of the absorbance at 570 and 690nm. Results presented as mean ± SEM (*n* = 4). *P* > 0.05 one-way ANOVA.

DEP exposure caused a dose-dependent increase in intracellular ROS in MEFs (Figure 2A) while exposure to urban dust SRM1649a, summer and winter TSP failed to do so (Figure 2A and B). The difference in fluorescence between DEP and urban dust-treated MEFs was statistically significant at concentrations from 0.006 to 0.05mg/ml (Figure 2A). H_2_O_2_ caused a dose-dependent increase in intracellular 2′,7′-dichlorofluorescein (DCF) fluorescence after 45-min incubation (data not shown). Exposure of DCFH_2_-DA-loaded MEFs to the highest concentrations of H_2_O_2_ tested for 2h or PM did not decrease cell viability compared to untreated DCFH_2_-DA-loaded cells as assessed using the MTT assay (Figure 2C).

**Figure 3.**
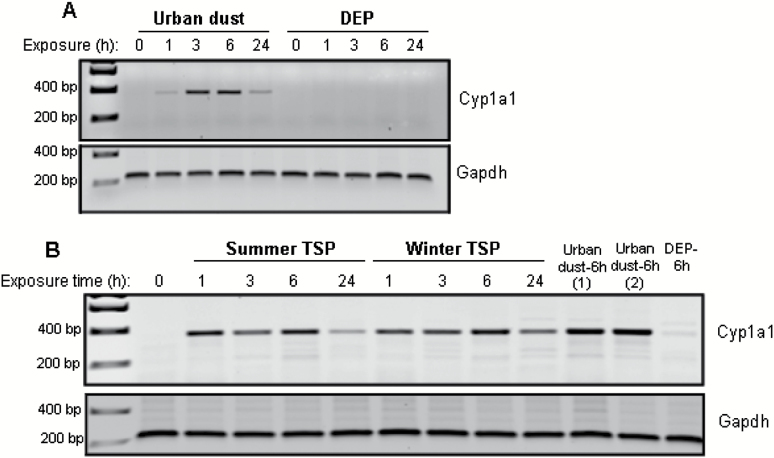
Expression of CYP1A1 mRNA in MEFs following PM exposure. MEFs were treated with sublethal dose of PM (**A**: urban dust SRM1649a and DEP, **B**: summer and winter TSP) (20 µg/ml) for 1–24h prior to RNA extraction. RT–PCR was carried out using specific CYP1A1 and glyceraldehyde 3-phosphate dehydrogenase (GAPDH) primers. The PCR products were separated by electrophoresis on 1.5% agarose gels and visualised on a multimode imager. CYP1A1 and GAPDH cDNA fragments amplified by PCR were 400 and 220bp, respectively.

We hypothesised that PM-associated PAHs may induce the expression of CYP450 enzymes required for PAH metabolism, which in turn may be a source of intracellular ROS. We therefore studied the effect of PM exposure on CYP1A1 mRNA levels in MEFs. The level of CYP1A1 mRNA increased as early as 1h after exposure to urban dust and PM and remained elevated 24h following exposure (Figure 3A and B). In contrast, little to no CYP1A1 cDNA could be amplified in DEP-treated MEFs at the concentration tested at any of the time points (Figure 3A). Thus, CYP1A1 gene expression was not linked to increase in intracellular ROS as detected using DCFH_2_-DA assay.

### Cytotoxicity of PM in MEFs

**Figure 4.**
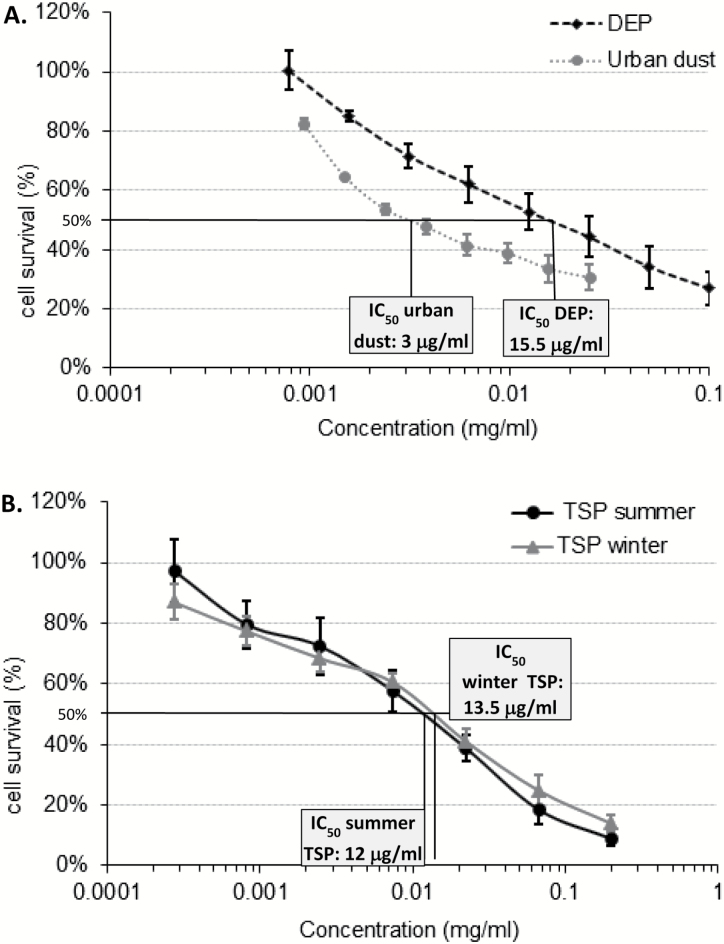
Cytotoxicity of urban dust (SRM1649a) and DEP (**A**) and Manchester TSP (**B**) in MEFs. MEFs were plated in 96-well plates and allowed to settle for 24h prior to addition of PM-containing medium in triplicate wells for a further 48h. PM-containing medium was subsequently replaced by medium containing 0.6mg/ml MTT and incubated for 4h. Formazan precipitate was dissolved in DMSO. Absorbance at 570 and 690nm was measured in each well. The proportion of cells that survived treatment compared to untreated cells was calculated at each concentration. Results are presented as mean ± SEM (*n* = 3).

The effect of PM on cell viability/survival was then investigated using the MTT assay. A dose-dependent decrease in cell survival was observed following exposure to all PM samples (Figure 4A and B). The average concentration inhibiting the growth of MEFs by 50% (IC_50_) compared to untreated cells for each PM was calculated from all MTT assay replicates. Urban dust 1649a exhibited the highest cytotoxicity of all samples analysed. Indeed, the IC_50_ of urban dust 1649a was ~3 µg/ml (Figure 4A) that was between four and five times lower than DEP (15.5 µg/ml, Figure 4A), summer (12 µg/ml, Figure 4B) and winter PM (13.5 µg/ml, Figure 4B).

### DNA damage level, assessed by the alkaline comet assay, in MEFs following PM exposure

**Figure 5.**
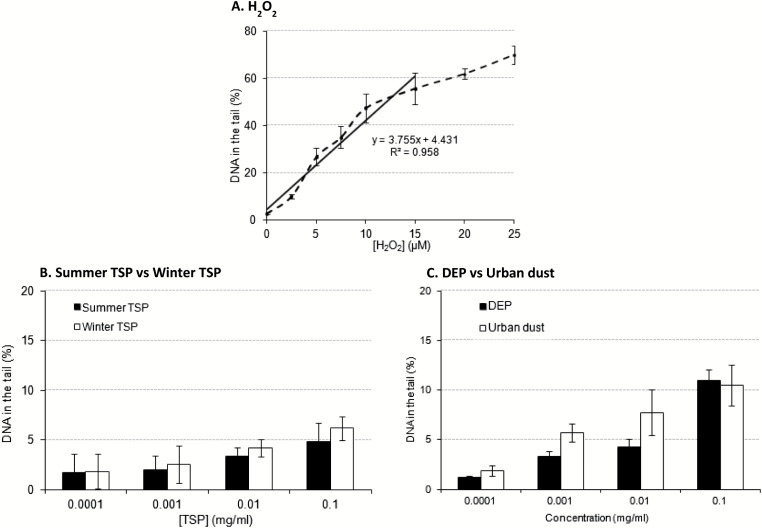
DNA damage, assessed by the alkaline comet assay, in MEFs induced by Manchester TSP (**A**), urban dust SRM1649a and DEP (**B**) and H_2_O_2_ (**C**). (A) MEFs (10^5^) were diluted in 150 µl PBS and 100 µl of 0–62.5 µM H_2_O_2_ (final concentration 0–25 µM) was added in each tube. MEFs were incubated for 10min on ice with H_2_O_2_ before the addition of 750 µl 1% LMP agarose (37°C). (B and C) MEFs (4×10^4^) were dispensed in wells of a 24-well plate and allowed to settle for 24h. The medium was replaced with medium containing 0–0.1mg/ml PM and incubated for 3h at 37°C. Cells were dissociated and cell pellets were suspended in 300 μl 0.7% LMP agarose (37°C). For all assays and conditions, two LMP drops were subsequently dispensed on agarose-coated glass microscope slides, a coverslip was added on each drop and the gels were left to set for 5min on ice. Cells were lysed for 1h at 4°C in lysis buffer and DNA was unwound for 40min at 4°C in electrophoresis buffer (pH > 13) prior to electrophoresis for 18min at 25V. Following neutralisation, the slides were stained with SYBR Green in the dark. Dry slides were stored in the dark until image capture using a fluorescent microscope and analysis with CometScore™. Results are presented as mean ± SEM (H_2_O_2_: *n* = 4, PM samples: *n* = 3).

The potential for PM-mediated DNA damage to contribute to the previously observed decrease in growth rate of MEFs was assessed using the alkaline comet assay. A DNA damage dose–response curve was established by treating MEFs with 0–25 µM H_2_O_2_ for 10min on ice, and results are shown in Figure 5A. There was a linear correlation between % DNA in the tail in MEFs and H_2_O_2_ concentrations ranging from 0 to 15 µM (Pearson correlation coefficient *R* ^2^ = 0.958). However, for H_2_O_2_ concentration above 15 µM (~55% DNA in the tail), the increase in % DNA in the tail levelled off and the DNA damage level reached saturation. There was evidence of dose-related increases in DNA damage following PM exposure (Figure 5B and C). At similar PM concentrations, urban dust and DEP exhibited higher levels of damage than summer and winter TSP.

### Genotoxicity of PM in the GreenScreen® Human Cell assay

**Figure 6.**
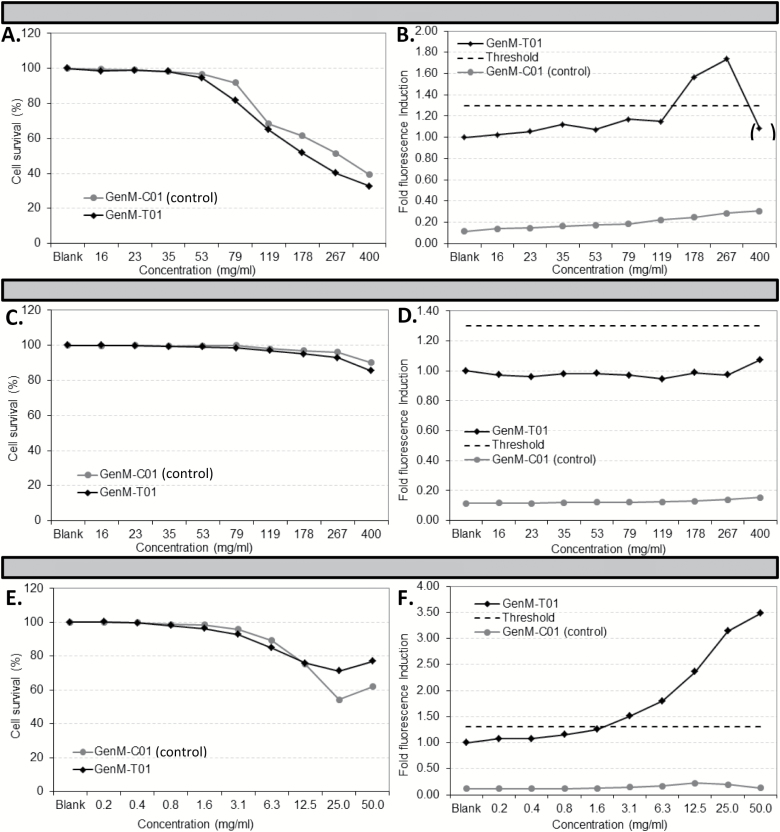
Cytotoxicity and genotoxicity of urban dust (SRM1649a; **A** and **B**) and DEP (**C** and **D**) assessed using the GreenScreen® HC assay. Duplicate serial dilutions of urban dust (SRM1649a), DEP and MMS (**E** and **F**: positive control) in 75 μl medium were established in a 96-well plate and 1.5×10^5^ of either reporter GenM-T01 or control GenM-C01 cells in 75 μl medium were added to each serial dilution. Following incubation for 48h at 37°C, PI was added across the plate and green and red fluorescence in each well was measured by flow cytometry. Cell viability was calculated as the proportion of PI-negative cells after treatment compared to PI-negative cells in control wells. The highest concentration of urban dust (in between brackets) did not satisfy the quality control requirement for a minimum of 10000 PI-negative events measurable by flow cytometry and results from GFP fluorescence analysis were thus voided. For other dilutions, fluorescence induction was calculated as the increase in fluorescence between treated and untreated reporter GenM-T01 cells. The threshold for positive genotoxicity is set at three times the standard deviation of the mean fluorescence intensity from samples treated with toxic and non-toxic non-genotoxicants (broken line). Data obtained with solvent-vehicle (DMSO) control are not shown.

We finally studied the ability of PM to induce the expression of GADD45a, one of the key responders to genotoxicant-induced stress response in p53-competent mammalian cells. Urban dust SRM1649a was cytotoxic in both GenM-C01 (control) and GenM-T01 cell lines (Figure 6A) but caused an increase in fluorescence (superior to the set threshold value) within the acceptable toxicity range of the assay and in reporter GenM-T01 cell line only, for concentrations >178 μg/ml (Figure 6B), i.e. the sample itself was not fluorescent. The highest concentration of urban dust used resulted in a 70% decrease in cell viability compared to untreated cells, leaving too few viable cells to fulfil the quality control requirements for flow cytometry analysis hence that result was voided. DEP showed little/no cytotoxicity (Figure 6C) in TK6 cells and no detectable GFP fluorescence induction above the threshold was seen (Figure 6D). MMS was used as positive control: it was cytotoxic in both TK6 cell lines (Figure 6E) and also induced an increase in green fluorescence in reporter GenM-T01 cells only (greater than the threshold) at concentrations >3.1 μg/ml (Figure 6F). Summer and winter TSP could not be analysed in this assay due to the limited amount of sampled material available at that stage.

**Figure 7.**
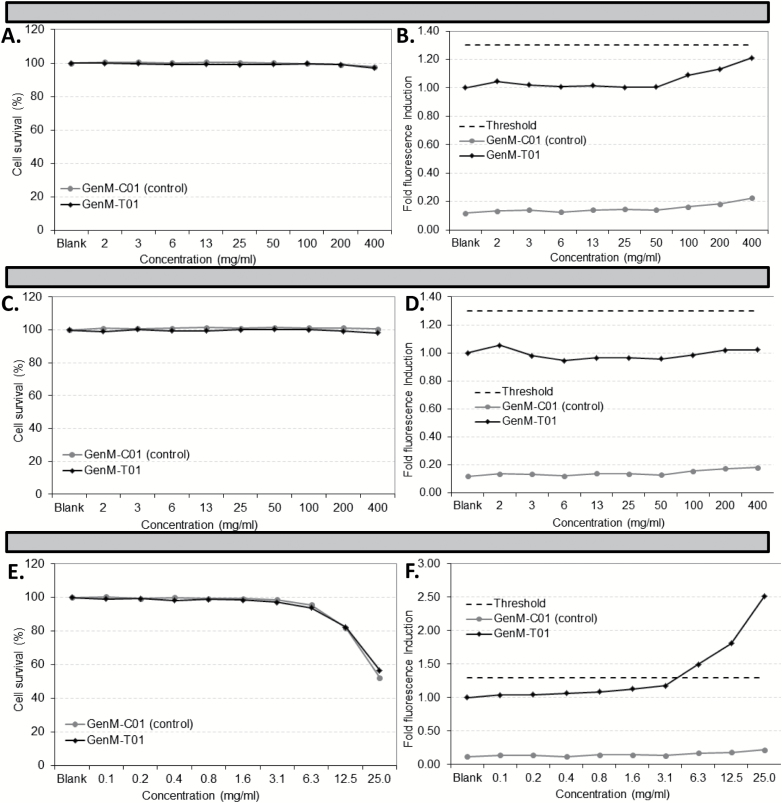
Cytotoxicity and genotoxicity of metabolically activated urban dust SRM1649a (**A** and **B**) and DEP (**C** and **D**) in GreenScreen® HC assay. Duplicate serial dilutions of urban dust (SRM1649a), DEP and cyclophosphamide (**E** and **F**: positive control) in 75 μl medium were established in a 96-well plate. A total of 1% (v/v) S9 rat liver extract was added in each well. Either reporter GenM-T01 or control GenM-C01 TK6 cells (1.5×10^5^) in 75 μl medium were then added in one serial dilution duplicate and incubated for 3h at 37°C with PM and S9 extracts. Cells were harvested by centrifugation, washed in PBS and resuspended in 150 μl serum-free medium and incubated for 45h at 37°C. PI was added in each well, and green and red fluorescence was measured by flow cytometry. All data points satisfied the requirement for 10000 viable observations. Cell survival was calculated as the proportion of PI-negative cells following treatment compared to viable cells in untreated wells. Fluorescence induction was calculated as the increase in fluorescence between treated and untreated GenM-T01 cells. A threshold for positive genotoxicity results was set at three times the standard deviation of the mean fluorescence intensity in control GenM-T01 cells (broken line).

Metabolic activation of urban dust and DEP using S9 extracts did not cause any decrease in cell viability (Figure 7A and C) and failed to induce any increase in GFP fluorescence past the threshold value in reporter GenM-T01 cell line (Figure 7B and D). S9-activated cyclophosphamide was cytotoxic in both TK6 cell lines (Figure 7E) and caused a subsequent increase in green fluorescence level (above set threshold value) in reporter GenM-T01 cells only for concentrations >6.3 μg/ml (Figure 7F).

## Discussion

In this study, the cytotoxicity and genotoxicity of PM samples collected from a busy urban thoroughfare in Manchester (UK) were examined and compared to that obtained with standard urban dust particulate sample (SRM1649a) and DEPs. All PM samples exhibited some levels of cytotoxicity and genotoxicity: urban dust (SRM1649a) was the most cytotoxic of the four samples, while the other three PM samples were of lesser but comparable potency (MTT assay). This was also confirmed by the GreenScreen Human Cell (HC) assay results where urban dust SRM1649a exhibited a much greater toxicity than DEP on control and reporter TK6 cell lines. Urban dust (SRM1649a) and DEP caused more strand breaks and alkaline-labile sites than summer and winter TSP samples (see summary Table 2). Despite earlier reports (6), the cytotoxic and genotoxic properties of PM used in our study did not clearly segregate with various direct and indirect measures of oxidative potential, including:

(i) elemental composition of the samples (as DEP and TSPs were of similar composition but had different potency in cytotoxicity assays),
(ii) acellular ROS production (as summer TSP catalysed the Fenton reaction to a greater extent than the other samples but exhibited relatively low levels of toxicity),
(iii) intracellular ROS production (as DEP was the only sample that resulted in the production of detectable levels of intracellular ROS but exhibited the lowest potency in terms of cytotoxicity of all samples tested),
(iv) CYP1A1 induction (as urban dust SRM1649a, summer and winter TSP but not DEP-induced CYP1A1 expression in MEFs).

Taken together, these results suggest that while intracellular ROS production can be an important determinant of cellular toxicity, other factors may also influence PM-mediated toxicity.

A variety of different inorganic and organic chemicals can be adsorbed onto the surface of PM, with transition metals and PAHs being of particular interest with regards to PM-induced toxicity. Transition metals can stimulate the production of ROS via the catalysis of the Fenton reaction while intracellular metabolism/detoxification of PAHs can form reactive intermediates that react with DNA and other macromolecules. Furthermore, quinones and semi-quinones formed during PAH metabolism can undergo redox cycling, also leading to ROS production. In this study, all PM samples could catalyse acellular H_2_O_2_ degradation via the Fenton reaction and subsequent plasmid DNA degradation in a dose-dependent manner, with summer TSP being the most potent inducer. While particle size has been shown to be an important determinant of DNA strand breakage induced by urban PM (40), it is unlikely that the limited differences in median particle size between PM samples can explain the differences in acellular ROS generation between PM samples documented in this study. The iron content of PM samples could also constitute an important factor with regards to Fenton-mediated ROS generation. Summer TSP exhibited the highest level of elemental iron of all PM samples analysed (Table 1) and this may explain results obtained in the acellular ROS assay: higher levels of metals in summer samples and enhanced cytotoxicity have previously been reported (41). However, in contrast to previous reports (39), none of the tested PM samples could induce a significant amount of plasmid DNA strand breakage in the absence of any redox active precursor (i.e. H_2_O_2_), which may reflect differences in PM composition and properties.

In contrast to these *in vitro* results, only DEP showed a clear dose-dependent increase in intracellular ROS levels in MEFs consistent with previous studies (42,43). Although the organic component of the PM samples was not characterised in this study due to limited sample availability, DEP samples typically contain less heavy metals and more PAHs and redox cycling quinones than urban PM [between 20 and 72 times more reactive quinones than urban dust 1649a: (6,44,45)]. This led us to hypothesise that intracellular ROS production in MEFs following DEP exposure may be mostly due to metabolic transformation of the organic component of PM rather than Fenton reaction catalysis by transition metals. To further investigate this hypothesis, the level of CYP1A1 mRNA in MEFs following exposure to a sublethal dose of PM was assessed, as CYP enzyme induction has previously been linked with increased intracellular ROS levels (46,47). Urban dust, summer and winter TSP caused an increase in CYP1A1 mRNA level consistent with previous studies (16,48,49), but despite evidence in the literature that some DEP samples (i.e. SRM from NIST) can induce CYP1A1 expression and activity (7,50), there was little evidence of this in our study. We cannot rule out changes in the expression of other genes but it is interesting that a rat liver (S9) extract failed to affect cell proliferation and stimulate a noticeable genotoxicant-induced stress response in GreenScreen HC assay using either urban dust SRM1649a or DEP.

Similar discrepancies between acellular and cellular ROS-generating capacity have been previously reported in the literature: indeed, the water-soluble fraction of PM_2.5_ generated free radicals (as detected by electron spin resonance) *in vitro* but did not result in significant intracellular ROS production (51). The presence of an efficient antioxidant network and varied metal-binding proteins in mammalian cells to control the reagents and products of the Fenton reaction may explain why the acellular ROS-generating capacity of PM does not correlate well with intracellular ROS levels (52). In addition, the sensitivity of acellular and intracellular ROS detection assays to the different ROS produced may differ, as doubts have been cast in the past about the sensitivity of DCFH_2_-DA oxidation assay to hydroxyl radicals, typically produced by the Fenton reaction (53).

Urban dust (SRM1649a) was found to be consistently more cytotoxic in MEFs and TK6 cells (GreenScreen HC assay) than the other PM samples examined in this study. Previously, urban dust was found to decrease human bronchial epithelial cell proliferation to a greater extent than DEP (54), without affecting cell viability as assessed by PI uptake (53), trypan blue uptake or alamar blue staining (44,54). The experimental conditions used for the MTT assay in our study (i.e. initial low cell density, relatively long incubation time with PM) however did not allow a clear distinction between a decrease in cell viability and a decrease in cell proliferation rate. Another study however reported that DEP was more cytotoxic and inhibited colony-formation ability to a greater extent than urban dust in A549 cells (6). These inconsistencies between studies highlight the fact that the toxicity of a given PM sample is intimately linked to its chemical and gravimetrical composition (6,29,41) as well as the cell system used for toxicity study, thus hampering meaningful cross-study comparison.

Despite the fact that only urban dust SRM1649a could induce GADD45a-mediated genotoxicant stress response, this process is unlikely to be triggered by DNA damage (as assessed by the alkaline comet assay) as there was little difference between DNA damage in mammalian cells exposed to either DEP or urban dust SRM1649, which is consistent with a previous report (6). In addition, our study failed to demonstrate that cytotoxicity and oxidative potential of PM could predict the level of DNA damage, assessed by the alkaline comet assay, following PM exposure, in contrast to a previously reported study (6). The response elicited in the GreenScreen HC assay by urban dust SRM1649a is also unlikely to be linked to intracellular ROS-generating capacity, suggesting that a ‘non-oxidative’ molecular pathway may be involved in urban dust SRM1649a genotoxicity. This was further investigated using the GreenScreen HC + S9 activation assay. Owing to differences in protocols and dilution of PM samples in S9 extracts, comparison of a particular PM sample across both types of assays is difficult; however, it seems that metabolic activation of PM is not a prerequisite to urban dust SRM1649a-induced genotoxicity.

In conclusion, although PM used in these studies can generate ROS in acellular systems and DEP, in particular, in mammalian cells, cytotoxic and genotoxic assays are not consistent with ROS production being the sole determinant of PM-induced toxicity. This suggests that the organic component can contribute significantly to this toxicity and that further work is required to better characterised the extent to which ROS and organic components contribute to PM-induced toxicity.

## Funding

This work was supported by an MRC scholarship to A.F.D.-V.

Conflict of interest statement: R.W. is the Founder and Scientific Director of Gentronix Ltd, which developed and sells the GADD45a-GFP GreenScreen HC genotoxicity assay. M.T. is employed by Gentronix Ltd.
